# Thermotolerance adaptation to human-modified habitats occurs in the native range of the invasive ant *Wasmannia auropunctata* before long-distance dispersal

**DOI:** 10.1111/eva.12058

**Published:** 2013-03-11

**Authors:** Julien Foucaud, Olivier Rey, Stéphanie Robert, Laurent Crespin, Jérôme Orivel, Benoit Facon, Anne Loiseau, Hervé Jourdan, Martin Kenne, Paul Serge Mbenoun Masse, Maurice Tindo, Merav Vonshak, Arnaud Estoup

**Affiliations:** 1 INRA, UMR1062 CBGPMontpellier, France; 2 CIRAD, UMR BGPI, Campus International de BaillarguetMontpellier, France; 3 INRA, UR346 d'Epidémiologie Animale, Université de LyonLyon, France; 4 CNRS, UMR5558, Laboratoire de Biométrie et Biologie Evolutive, Université Lyon 1Villeurbanne, France; 5 CNRS, UMR Ecologie des Forêts de Guyane, Campus AgronomiqueKourou cedex, France; 6 IMBE, Aix-Marseille University, Centre IRD de Nouméa,Nouméa, New Caledonia; 7 Département de biologie des organismes animaux, faculté des sciences de l'université de DoualaDouala, Cameroun; 8 Laboratoire de Zoologie, Faculté des Sciences, Université de Yaoundé,Yaoundé, Cameroun; 9 Department of Biology, Stanford UniversityStanford, CA, USA

**Keywords:** adaptation, heat shock, invasive species, natural selection and contemporary evolution, thermotolerance

## Abstract

Key evolutionary events associated with invasion success are traditionally thought to occur in the introduced, rather than the native range of species. In the invasive ant *Wasmannia auropunctata*, however, a shift in reproductive system has been demonstrated within the native range, from the sexual non-dominant populations of natural habitats to the clonal dominant populations of human-modified habitats. Because abiotic conditions of human- modified habitats are hotter and dryer, we performed lab experiments on workers from a set of native and introduced populations, to investigate whether these ecological and genetic transitions were accompanied by a change in thermotolerance and whether such changes occurred before establishment in the introduced range. Thermotolerance levels were higher in native populations from human-modified habitats than in native populations from natural habitats, but were similar in native and introduced populations from human-modified habitats. Differences in thermotolerance could not be accounted for by differences in body size. A scenario based on local adaptation in the native range before introduction in remote areas represents the most parsimonious hypothesis to account for the observed phenotypic pattern. These findings highlight the importance of human land use in explaining major contemporary evolutionary changes.

## Introduction

Most recent studies trying to decipher the reasons for the success of invasive species have explicitly focused on the introduced range of invasive species (Richardson et al. 2000; Sakai et al. 2001; Colautti and MacIsaac 2004). This is logical given that post-introduction ecological and evolutionary challenges are likely to be more important than those occurring before long-distance dispersal. After their introduction into a new area, founding individuals generally have to cope with new demographic and/or environmental conditions when compared to their native habitats. New demographic conditions, such as Allee effects or genetic drift in small populations, can hamper successful establishment (Sakai et al. 2001). New biotic and abiotic conditions can also constitute severe barriers to successful invasions (Blackburn et al. 2011). Comparisons between native and introduced populations of invasive species implicitly seek to unravel the evolutionary changes that may have occurred in the introduced range (Keller and Taylor 2008; van Kleunen et al. 2010).

However, critical biological switches can also occur within the native range of invasive species (Bossdorf et al. 2008; Lee and Gelembiuk 2008). The role of preadaptation (or prior adaptations, see Hufbauer et al. 2012) of certain native populations to explain invasive success has not received adequate attention (van Kleunen et al. 2011), despite early studies underlying this possibility (Elton 1958). In particular, native populations adapted to human- modified habitats may be particularly prone to become invasive elsewhere (Ehrlich 1989). Human activities are now widely recognized as a major force promoting biological invasions (e.g. King and Tschinkel 2008; Leprieur et al. 2008), both through large-scale transport (Floerl and Inglis 2005; Suarez et al. 2005; Tatem et al. 2006) and biotic homogenization (e.g. McKinney and Lockwood 1999; Olden et al. 2004; McKinney 2006). Human activities provide native species with an opportunity to cross habitat boundaries locally, and to adapt to human- driven biotic and abiotic conditions within their native range (i.e., pre-adapt). In addition to triggering a general decrease in species abundance, functional diversity and other biotic effects (Olden et al. 2004; Ekroos et al. 2010), human activities tend to alter the abiotic properties of habitats, such as soil chemical and physical features, water resources and properties, air filtering, light incidence, and climatic conditions (Pickett et al. 2001; Kozlov and Zvereva 2007; Hrodey et al. 2009). Native populations adapting locally to such profound changes to their environment may subsidiarily benefit from the opportunity for long-distance dispersal provided by human activities. For instance, the worldwide airline transportation network provides numerous high capacity routes between geographically distant, but climatically similar locations (Tatem and Hay 2007). Native species that adapted to human- modified habitats are thus likely to access new areas characterized by similar habitat alterations, potentially multiple times, following human-driven introduction events (e.g. McKinney 2006; Foucaud et al. 2010; Hufbauer et al. 2012).

Although the idea of human activities promoting invasion success through prior adaptations in the native range is not new (Elton 1958; Ehrlich 1989), a renewed attention has been turned to the study of the native range and evolutionary changes occurring before introduction (Lee and Gelembiuk 2008; Valéry et al. 2009; Schlaepfer et al. 2010; Hufbauer et al. 2012). However, this interest has been mostly theoretical, and the importance of prior adaptations to human-modified habitats in generating successful invaders has seldom been studied using natural populations (van Kleunen et al. 2010). Notable exceptions are studies comparing life-history traits between native populations of invasive and non-invasive species (Chown et al. 2007; Schlaepfer et al. 2010; Jenkins and Keller 2011; van Kleunen et al. 2011), or population history of native populations differing in their physiological ability to invade novel habitats (Winkler et al. 2008). These studies all underline the importance of prior adaptations to human-modified, marginal, or fluctuating habitats within the native range in explaining successful invasions events. However, the relative contribution of prior adaptation and other processes (multiple introductions, post-introduction adaptation) to invasion success remains speculative, given the scarcity of experimental studies of native populations of invasive species.

*Wasmannia auropunctata,* an invasive ant species ranked among the most destructive invaders worldwide (Lowe et al. 2000), presents the opportunity to test for the hypothesis of important adaptations occurring within the native range before long-distance introductions. Previous studies have shown that this ant underwent major biological shifts within its native range before the long-distance dispersal events leading to worldwide invasion. In natural areas of its native range (primary forests of South America), *W. auropunctata* forms low-density populations (Orivel et al. 2009). However, this ant has also successfully colonized human- modified habitats within its native range, and this habitat change was paralleled by a striking switch in ecological dominance (but not in social system; Foucaud et al. 2009; Orivel et al. 2009). A switch in reproductive system also took place at the time of habitat transition: primary forest populations generally display a classical haplo-diploid reproductive system (hereafter referred to as ‘sexual’), whereas populations from human-modified areas are almost entirely clonal, with a strong reduction in genetic variability (Foucaud et al. 2007). All introduced populations studied to date appear to be drawn from dominant populations of human-modified habitats of the native range of the species (Foucaud et al. 2010), suggesting that ecological and reproductive shifts occurred within the native range before long-distance dispersal.

We have previously shown that abiotic conditions differ considerably between the natural and human-modified environments of *W. auropunctata* in its native range (Orivel et al. 2009). Over the course of the year, temperature remains stable at values below 30°C and humidity never drops below 80% in natural habitats, whereas in human-modified habitats, temperatures may reach 40°C and humidity may drop to 50%. The worker caste, in charge of the foraging outside the colony, may be particularly affected by these conditions. These abiotic differences clearly constitute a major ecological challenge, which has apparently been met by some native *W. auropunctata* populations. However, it remains unknown whether populations from natural and human-modified habitats differ in their ability to tolerate hot and dry abiotic conditions (hereafter referred to as ‘thermotolerance’, *sensu lato*), and whether putative differences in thermotolerance stem from local adaptation (Dybdahl and Kane 2005; Preisser et al. 2008) or phenotypic plasticity (Chown et al. 2007; Nyamukondiwa et al. 2010).

If both native and introduced populations from human-modified habitats can tolerate such conditions, and native populations from natural habitats cannot, this would support the scenario of an evolutionary shift in the native range before long-distance dispersal into the introduced range. This has previously been shown to occur for lower thermal limits of *W. auropunctata*, with a prior adaptation to cold in populations from Argentina, which allowed a successful remote introduction of the species in the Mediterranean zone (Rey et al. 2012).

A possible way for certain populations to tolerate difficult abiotic conditions could be to display variation in worker body size. Worker body size has been shown to play an important role in thermotolerance in several ant species (Kaspari 1993). In particular, *Cataglyphis velox* workers, desert ants that have to deal with extreme heat, have been demonstrated to make use of their large body size to forage at higher temperatures (Cerda and Retana 1997, 2000). In *W. auropunctata*, previous studies have shown that workers from populations established in the introduced range are smaller than those found in the native range (McGlynn 1999; Mikheyev and Mueller 2007). However, these studies did not compare the body sizes of native and introduced workers originating from populations established in different habitats (i.e., natural versus human-modified habitats, as in this study).

The main goal of this study was to investigate a putative evolutionary shift within the native range before long-distance dispersal that may indicate prior adaptation of certain native populations to become invasive. We tackled this question by testing in the laboratory the putative differences in upper thermotolerance levels between a set of native populations from natural and human-modified habitats and introduced populations from human-modified habitats. All tested populations have been genetically characterized at microsatellite markers to assess the genetic relationships among them and among other worldwide populations. We further investigated whether observed thermotolerance differences were due to body size variations. The possibility of prior adaptation to human-modified habitats within the native range in explaining invasive success is finally discussed.

## Materials and methods

### Sampling of live populations and setting of laboratory colonies

**Figure 1:**
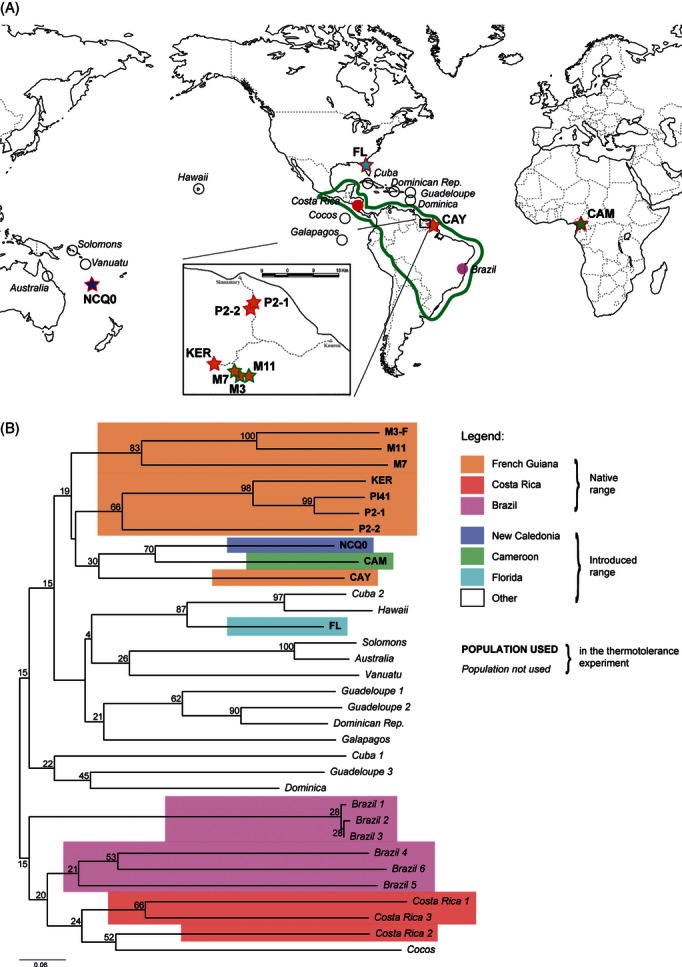
Sampled native and introduced populations of *W. auropunctata* and their genetic relationships. (A) Map of the sampled *W. auropunctata* populations. Populations that were used in the thermotolerance experiment are indicated with stars, filled with color representing their sampling location as in (B). Green- and red-lined stars indicate natural and human-modified habitats, respectively. Circles indicate populations that were only included in the genetic analyses. (B) Neighbor-Joining dendrogram of the microsatellite (allele-shared) distances between worldwide *W. auropunctata* populations. All bootstrap values are provided but note that low bootstrap values (<50%) occur at some basal nodes, precluding genetic proximity to be inferred based only on this reconstruction. The dendrogram is not rooted.

We collected ca. 140 queens and several thousand workers from 11 populations from natural and human-modified habitats within the native range (i.e., French Guiana) and the introduced range (i.e., Cameroon, Florida and New Caledonia) of *W. auropunctata* (Fig. 1, Table 1) between December 2007 and March 2008.

**Table 1:** Sampling design for native and introduced *W. auropunctata* populations. The locations of the sampling sites and their names correspond to previously studied sites (see Fournier et al. 2005a; Foucaud et al. 2007, 2009)

Range	Region	Population	Habitat
Native	French Guiana	M3-F	Natural
Native	French Guiana	M7	Natural
Native	French Guiana	M11	Natural
Native	French Guiana	Ker	Human-modified
Native	French Guiana	P2-1	Human-modified
Native	French Guiana	P2-2	Human-modified
Native	French Guiana	Cay	Human-modified
Native	French Guiana	Pi41	Human-modified
Introduced	Florida	Fl	Human-modified
Introduced	Cameroon	Cam	Human-modified
Introduced	New Caledonia	NCQ0	Human-modified

On the basis of our previous genetic studies of the sampled populations, we expected three of the sampled populations to be sexual and eight to be clonal (Foucaud et al. 2006, 2007). This was confirmed by genotyping a minimum of three queens and 30 workers from each population at 33 microsatellite loci, as described in Fournier et al. (2005b) and Almany et al. (2009). The reproductive system was determined by visually inspecting genotypes and using a program to identify identical multilocus genotypes that we had developed in Pascal object programming language (available upon request to the authors; see Foucaud et al. 2007; Fournier et al. 2005a).

All the populations sampled from natural habitats were sexual, whereas all the populations sampled in human-modified habitats were clonal: the factors ‘habitat’ and ‘reproductive system’ are thus confounded. In previous studies (e.g. Foucaud et al. 2007), we encountered, on very rare occasions, clonal populations in natural habitats (only twice in French Guiana) and sexual populations in human-modified habitats (only once in French Guiana). As these types of populations are extremely rare and difficult to sample, we were unable to include them in this study. Furthermore, clonal populations from natural habitats have so far been sampled only from the canopy, whereas sexual populations are found only at ground level. It therefore seems likely that clonal and sexual populations from natural habitats do not share the same ecological niche. We were thus unable to assess the interaction between habitat and reproductive system in this study, essentially because it was seldom present in the field.

We set up 11 laboratory colonies (Table 1), consisting of more than ten queens and at least 500 workers originating from three to 10 nests sampled in locations known to harbor a single population (Foucaud et al. 2009). We could gather many queens in each of the laboratory colonies because both sexual and clonal populations from the whole range of *W. auropunctata* are polygynous (i.e., no transition from monogyny to polygyny occurred in the course of the invasion process; Foucaud et al. 2009; Orivel et al. 2009). These laboratory colonies were kept in a climate chamber at a constant temperature of 25°C, with a humidity of 60% to 70%. Food (sugar water and worms) and water were provided *ad libitum* once per week.

### Genetic relationships between populations tested for thermotolerance

To investigate the putative adaptive evolution of a trait, it is important to compare populations sharing sufficiently close genetic relationships (e.g. Hufbauer et al. 2012). Previous genetic studies demonstrated that all sampled native populations from human- modified habitats originated recently from sampled native populations from natural habitats (Foucaud et al. 2007).

Previous genetic studies of worldwide introduction routes did not enable to directly link any introduced population to its exact native source population (Foucaud et al. 2010). This is mostly due to the strong genetic structure uncovered in the native range of the species (Foucaud et al. 2007). To validate the comparison between the three tested introduced populations (Cameroon, Florida, New Caledonia) and the tested native populations (French Guiana), we investigated whether these introduced populations were genetically closer to three alternative sets of native populations from Brazil, Costa Rica or French Guiana. To this aim, we genotyped at least 30 individual workers per sampled native and introduced population at 12 microsatellite markers (Fournier et al. 2005b) and added this new data set to a previous data set investigating the worldwide genetic variation of *W. auropunctata* (Foucaud et al. 2010).

We performed three distinct population genetics analyses using this global microsatellite data set. We first used the software populations (Langella 2002) to construct a dendrogram connecting worker populations using the Neighbor-Joining (NJ) algorithm (Saitou and Nei 1987). The genetic distance used was Chakraborty and Jin's allele- shared distance (Chakraborty and Jin 1993). This distance was averaged over all pairs of individuals belonging to pairwise populations in order to obtain the genetic distances between populations. Bootstrap values (with the locus as a unit) were calculated over 1000 iterations. Extended majority rule was applied to reconstruct the consensus tree. Similar results were obtained using other genetic distances (including Nei's chord distance, Cavalli-Sforza & Edwards distance and the proportion of shared alleles; Bowcock et al. 1994; Cavalli-Sforza and Edwards 1967; Nei et al. 1983) and reconstruction algorithms (UPGMA and BioNJ; Gascuel 1997). These additional phylogenetic reconstructions were obtained using MSA 4.05 for the computation of 10 000 bootstrapped distance matrices (Dieringer and Schlötterer 2003) and the neighbor and consense programs of the phylip package v3.69 for the tree reconstruction (Felsenstein 2004). As second analysis, we computed a matrix including pairwise *F*_ST_ values between all sampled populations using the estimator θ of the parameter *F*_ST_ (Weir and Cockerham 1984). We also performed an amova analysis using Arlequin 3.5 (Excoffier and Lischer 2010) to measure the partition of genetic variance between the introduced populations collected for the thermotolerance experiments and each of the available native populations (Brazil, Costa Rica and Guiana). As third analysis, we performed an exclusion analysis using GeneClass2 (Piry et al. 2004) to further investigate which native populations were genetically the most likely sources of the individual genotypes of the sampled introduced populations collected for the thermotolerance experiments.

### Thermotolerance assays

We assessed the tolerance of the workers to abiotic conditions in a resistance test in which mortality rate was determined in groups of workers. We placed ten individual workers in a Petri dish sealed with a grid cover and maintained the workers for 3 h at constant temperature and humidity conditions within a climate chamber. The number of dead workers at the end of this 3-h period was used as a measurement of the thermotolerance (*sensu lato*, i.e., including both tolerance to high temperature and to low hygrometric conditions). Mortality was assessed visually by four experimenters that were randomized according to treatments and populations tested.

Workers from laboratory colonies were tested more than 40 days after sampling, to ensure that all the workers tested had been produced in the laboratory and not sampled from the field (the life expectancy of *W. auropunctata* workers is about 30 days, (Ulloa-Chacon 1990). This time lag to the start of the experiments minimized the impact of the acclimation factor in the interpretation of our results. In addition, evaluation of the impact of acclimation on the measure of upper thermal limit in *Linepithema humile* shows that in our case of 25°C acclimation temperature and a high rate of temperature change, no acclimation effect should be expected (Chown et al. 2009). Similar experiments carried out on workers from colonies housed in the laboratory for more than a year provided qualitatively similar results, confirming that acclimation is unlikely to account for our findings (data not shown).

Preliminary experiments were conducted in the laboratory on one sexual population from a natural habitat (M7) and one clonal population from a human-modified habitat (Ker), to determine the range of temperature and humidity conditions yielding moderate to high worker mortality rates. We studied a total of 14 250 workers in 39 combinations of temperature (seven values from 25°C to 42°C) and humidity conditions (seven values from 100% to 25%; see Fig. S1 for details). On the basis of the findings of these preliminary experiments, we chose four temperature and humidity combinations giving moderate to high worker mortality rates for the main experiment: 36°C – 55%, 38°C – 65%, 39°C – 70% and 40°C – 75%. According to the preliminary data (Fig. S2), the chosen temperature- humidity combinations represent adequate conditions to detect differences in mortality between the two types of populations.

During the main experiment following our preliminary tests, we tested a total of 8790 workers as follows. For each of the four chosen sets of abiotic conditions, we tested all 11 laboratory colonies twice, using ten Petri dishes of ten individuals (i.e., 4 conditions * 11 colonies * ten Petri dishes * two runs = 880 Petri dishes, minus one missing). All eight assays were conducted in the same climate chamber, in which two Petri dishes per colony per run were placed at random positions on five vertical racks. As before, we recorded the proportion of dead workers per Petri dish (i.e., the mortality rate). We performed a control consisting in holding one additional Petri dish per tested laboratory colony (with 10 individual workers) at laboratory conditions (25°C – 60–70% RH) during the 3 h of each run, to monitor putative mortality due to the treatment itself.

### Statistical analysis of thermotolerance

Our main goal was to investigate putative differences in abiotic tolerance between populations of different origins (native natural, native human-modified and introduced human-modified), through the use of a statistical model. Explanatory variables consisted only in categorical factors. We used five categorical factors: ‘type’ (corresponding to the three different types of habitat), ‘pop’ (the sampled population), ‘cond’ (the tested temperature- humidity conditions), ‘rep’ (the number of the replicate, from one to two), and ‘rack’ (the vertical position of the Petri dish in the climate chamber, from rack 1 to rack 5). Note that ‘pop’ was nested within ‘type’ and that the variable ‘rep’ was nested within ‘cond’ (hereafter denoted ‘pop(type)’ and ‘rep(cond)’). Both the ‘pop’ and ‘rep’ factors were treated as random effects, whereas the remaining factors (‘cond’, ‘rack’ and ‘type’) were treated as fixed effects. For the purposes of interpretation, only the following interactions were included in the full model: cond*type, cond*pop(type), rack*pop(type), rack*rep(cond) and rep(cond)*pop(type). Given that the response variable was a proportion, we assumed a binomial distribution.

With fixed and random effects and a binomially distributed independent variable, we ran a generalized linear mixed model with a logit link function (Zuur et al. 2009). We were principally interested in estimating the magnitude of the difference in mortality rates between the three types of population, while correcting for several clustering factors (e.g., population). We therefore used Penalized Quasi-Likelihood methods (PQL; Breslow and Clayton 1993), as implemented in SAS 9.1, to fit the model (PROC GLIMMIX, SAS Institute, Cary, NC, USA). PQL methods may yield biased estimates of variance components in some situations (Lin and Breslow 1996). However, on the basis of previous work (Breslow and Clayton 1993; Breslow and Lin 1995; Bolker et al. 2009), we conclude that our estimates may only be subject to a negligible bias, given that (i) the data were only slightly unbalanced, (ii) all fixed effects reached high levels of significance, and (iii) all variance component estimates were small (<0.36) and the overdispersion coefficient was moderate (1.53, see below). The significance of fixed effects was assessed through approximate F-tests and the number of denominator degrees of freedom for each test was computed using the Kenward-Roger approximation (Bolker et al. 2009).

It is worth stressing that our full model yielded an estimate of 0 for the variance component of the interaction between replicate and vertical position within the climate chamber [i.e., ‘rack*rep(cond)’] and the interaction between the type of habitat and the conditions tested (i.e., ‘type*cond’) was clearly non-significant (*F*_[6, 25.8]_ = 1.15, *P =* 0.36). Therefore, as suggested by Zuur et al. (2009), we fitted a second model from which these two terms were removed. With this model, the coefficient of overdispersion was moderate (1.53 SE = 0.08), estimates of variance components were not large (i.e., ≪1; Lin and Breslow 1996) and all fixed effects other than the conditions tested (‘cond’) gave small *P*-values (see Table 2). This second model was therefore selected for subsequent analyses.

**Table 2:** Significance of fixed effects on the mortality rate of native and introduced workers of *W. auropunctata*. Degrees of freedom for the numerator and denominator are provided for each effect (columns Num DF and Den DF, respectively). Effect ‘Type’ corresponds to the three different types of habitat, ‘cond’ to the temperature-humidity conditions tested and ‘rack’ to the vertical position of the Petri dish in the climate chamber. See Materials and methods section for details

Effect	Num DF	Den DF	*F*-value	*P-*value
Type	2	7.876	14.84	0.0021
Cond	3	4.48	8.59	0.0257
Rack	4	38.21	37.2	<.0001
Cond*Rack	12	771.4	6.27	<.0001

### Effect of body size on thermotolerance

We tested for a possible effect of body size on our thermotolerance results, by investigating worker body size in five native populations – two from natural habitats (M3-F and M11) and three from human-modified habitats (Ker, P2-2 and Cay) – and two introduced populations (New Caledonia and Australia). The introduced Australian population, which was not tested in our main thermotolerance assay, has previously been shown to be clonal Foucaud et al. 2010, and subsequently to exhibit thermotolerance levels similar to those of other introduced populations (results not shown). We collected and point-mounted 20 foraging workers from each population. Multifocus images (x40) of worker heads were acquired with a GT Entovision System and ArchimedTM software (Microvision Instruments, Evry, France). Four morphological parameters of the head were measured on each worker (maximum head width, maximum head length, and head width in front of and behind the eyes) with Cartograph software (Microvision Instruments).

Preliminary analysis showed that all morphometric measurements were highly correlated (all Pearson's product-moment correlations > 0.66). Thus, as maximum head width is often considered a standard proxy for body size (Hölldobler and Wilson 1990), we used only this morphological variable for subsequent analyses. After testing for normality and variance homogeneity, we investigated putative differences in body size between populations originating from different habitats in both ranges (native natural, native human-modified, and introduced human-modified), by fitting a linear mixed model implemented in R (R Development Core Team 2005) and including the following factors: ‘type’ (fixed factor corresponding to our three types of population) and ‘pop’ (random factor corresponding to the sampled population, nested within ‘type’). We additionally investigated whether mean values of thermotolerance were related to mean body size at the population level for the six populations for which measurements were available for both traits. After checking for binormality of the data, we conducted a Pearson's product-moment correlation test using R.

## Results

### Genetic relationships between populations

The NJ tree presented in Fig. 1B shows that the collected native Guianese populations group together with the introduced populations used in our thermotolerance experiments (New Caledonia, Cameroon, and to a lesser extent Florida). The support for the deepest nodes of the tree was however low (bootstrap values below 20%), due at least partly to a too low number of microsatellite markers and an incomplete sampling given the substantial genetic structure at small geographic scale in the native range of this species Foucaud et al. 2009;. Low node values were also somewhat expected given the strong bottleneck events endured by clonal populations (i.e., an equivalent of only two effective individuals, one haploid male and one diploid female and hence a maximum of three alleles per locus), likely negatively influencing our ability to reconstruct a correct phylogeny (Takezaki and Nei 1996; Estoup and Guillemaud 2010). All distances and reconstruction algorithms however yielded similar topologies (results not shown).

The pairwise *F*_ST_ values calculated among all populations sampled in the distribution range of *W. auropunctata* showed that our focal introduced populations used for thermotolerance experiments were genetically closer to native Guianese populations than to any other sampled native populations (Brazil and Costa Rica; Fig. S2). In agreement with this, the amova analysis showed that the least amount of genetic variance was partitioned between the focal introduced populations and Guianese populations: 5.52% of genetic variation versus 11.52% and 12.44% for the Brazilian and Costa Rican populations, respectively.

Exclusion analysis results indicated that all native populations were statistically excluded as the source with a probability *P* < 0.01 for all individuals of our focal populations. This result suggests that we did not sample the exact source location of the studied introduced populations. It is worth noting, however, that in the case of an extreme reduction of genetic variability (which is the case in bottlenecked introduced *W. auropunctata* populations), the GeneClass2 algorithm may fail to assign individuals to their true source with a high probability due to strong drift effect. In agreement with the *F*_ST_ and amova results, the likelihood ratio scores provided by GeneClass2 for each introduced individual showed that the Guianese populations were however the most likely sources when compared to any other sampled native population (Brazil and Costa Rica).

Overall, our genetic analyses suggest that (i) although strong bottleneck events most likely substantially inflated genetic differentiation, it was unlikely that the sampled Guianese populations were the exact source(s) of the introduced populations tested for thermotolerance experiments, but that (ii) among available native populations (Brazil, Costa Rica and Guiana), all tested introduced populations were genetically closer to the (also tested) Guianese populations. Therefore, although the genetic relationships between the native and introduced populations were not fully resolved, the choice of Guianese populations to represent the native range constituted the best option available to test for an adaptive shift between *W. auropunctata* populations from the native and introduced ranges. In addition, a previous microsatellite study investigating the genetic relationships between individuals from Guianese native populations showed that sexual populations from natural habitats were the recent source of the clonal populations found in human-modified habitats (Foucaud et al. 2007). The present microsatellite data and statistical treatments confirmed this close genetic relationship and hence validate the phenotypic comparison of the sampled native populations to investigate a putative adaptive shift within the native range of *W. auropunctata*.

### Thermotolerance assays

**Figure 2:**
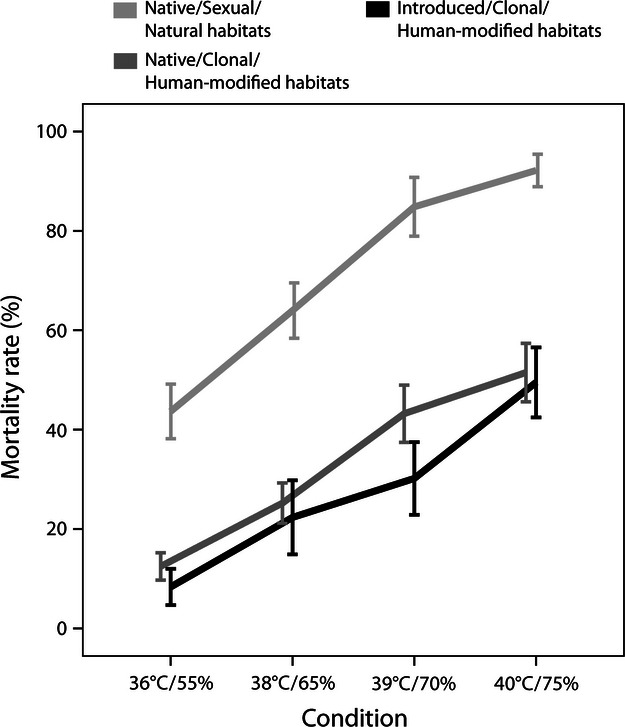
Mortality rates of workers from three *W. auropunctata* population types for each experimental condition of the thermotolerance assay. Populations types are sexual populations from natural habitats in the native range, clonal populations from human-modified habitats in the native range and clonal populations from human-modified habitats in the introduced range. Mortality rates are mean proportions of dead workers per group of ten workers placed in the same Petri dish. Error bars indicate 95% confidence intervals.

Mortality rate was significantly higher for workers of populations from natural habitats of the native range (NaN) than for workers of populations from human-modified habitats of the native (NaH) or introduced ranges (IntH) [mortality_NaN_ = 0.79, IC_95%_ *=* (0.60; 0.91); mortality_NaH_ = 0.29, IC_95%_ *=* (0.16; 0.47); mortality_IntH_ = 0.21, IC_95%_ *=* (0.09; 0.40)*; t*_NaN-NaH, 7.94_ = −4.97, *P* = 0.0011 and *t*_NaN-IntH, 7.85_ = −4.63, *P* = 0.0018; Fig. 2; Fig. S3 and S4]. In contrast, no significant difference was found between the mortality rates of the workers of populations from human-modified habitats of the native and introduced ranges (*t*_NaH-IntH,7.85_ = −0.95, *P* = 0.37).

We also found a significant condition effect (Fig. 2; Table 2), meaning that our four sets of conditions resulted in moderate to high mortality rates, as expected on the basis of our preliminary results (summarized in Fig. S1). The lack of interaction between the factors ‘type of population’ and ‘condition’ indicates a constant relationship between population type and mortality from moderate to harsh abiotic conditions (Fig. 2). However, without *a priori* power computations, we cannot discard definitively that these interactions existed and were not significant because of a lack of power. The vertical position of the Petri dish within the climate chamber had a significant effect on worker mortality rate (Table 2). This effect was expected, given the pitfalls of current climate chamber technology (i.e., the occurrence of vertical gradients), and was controlled through the equilibration of our experimental design (i.e., equal mixing of all populations at each vertical position within the climate chamber). Importantly, no mortality occurred in the control Petri dishes held at laboratory conditions (i.e., no difference in mortality between populations at laboratory conditions).

### Body size and thermotolerance

**Figure 3:**
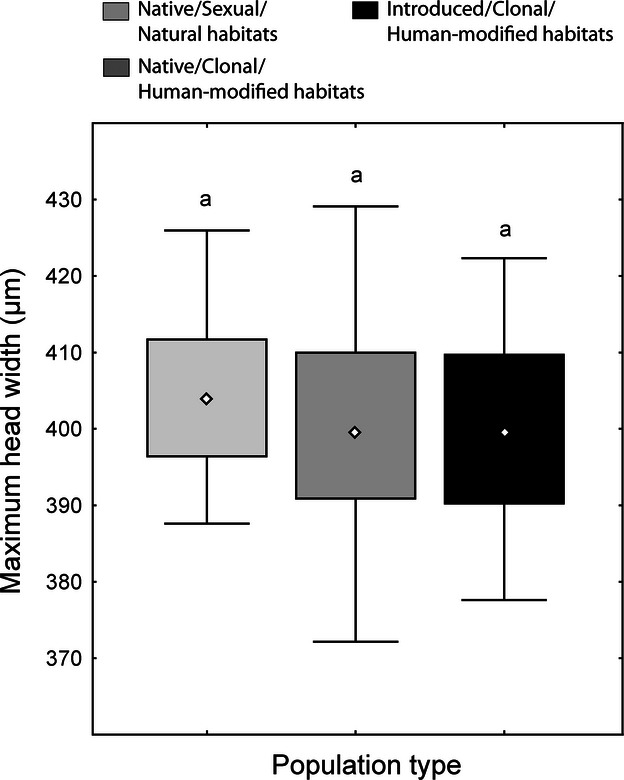
Maximum head width of workers from the three population types. Diamonds indicate means, blocks and horizontal bars indicate 50% and 95% percentiles, respectively.

Maximum individual head width was normally distributed (Shapiro–Wilk test: *W* = 0.989, *P* = 0.41) and individual variances were homogeneous for workers from different population types – that is, for workers from native natural, native human-modified, and introduced human-modified habitats (Brown-Forsythe Levene-type test: *P* > 0.09). The linear mixed model analysis revealed that the type of population factor was not significant (χ² = 0.6287, *P* = 0.73; Fig. 3). Workers thus had similar body sizes regardless of their habitat of origin (i.e., native natural, native human-modified, and introduced human-modified).

Furthermore, there was no linear relationship between mean values of thermotolerance and body size at the population level (Fig. S5). After checking for the binormality of the data, a Pearson's product-moment correlation test indicated no significant correlation between mean thermotolerance and body size values (*t* = 0.9471, df = 4, *P* = 0.3972).

## Discussion

### Adaptive shift between natural and human-modified habitats within the native range

Our thermotolerance study indicated that a major phenotypic transition likely occurred within the native range of *W. auropunctata*. Workers from native sexual populations sampled in natural habitats were less tolerant to hot and dry conditions than both native and introduced clonal populations sampled in human-modified habitats. In contrast, populations established in human-modified habitats in the native and introduced ranges displayed similar, higher levels of thermotolerance. The fact that native populations from natural and human-modified habitats share close genetic relationships indicate that an adaptive shift most likely occurred within the native range of *W. auropunctata*, before long-distance dispersal. On the other hand, the less robust genetic relationships observed between the sampled native and introduced populations make it more difficult to unambiguously conclude that the high level of thermotolerance of introduced populations derived from the prior adaptation of native Guianese populations in human-modified habitats.

A previous study has shown that climatic conditions differ considerably between adjacent natural and human-modified habitats in the native range of *W. auropunctata*, with the latter reaching higher temperatures and lower humidity (Orivel et al. 2009). Some of the abiotic conditions tested in our study correspond to the worst possible conditions found in the field during an 18-month period, rather than to conditions likely to be experienced on a regular basis by workers. However, the higher mortality rates for workers from native natural habitats populations in the tested abiotic conditions likely translate in a lower foraging efficiency and lower fitness in native human-modified habitats when compared to heat- tolerant populations. Consistent with this hypothesis, Cerdá et al. (1998) have shown that, in a Mediterranean ant community, heat-tolerant species have a higher foraging efficiency at high temperatures than heat-intolerant species.

In invasive organisms, including insects, it has been shown that wider thermotolerance abilities can stem either from local adaptation (e.g., in the snail *Potamopyrgus antipodarum*, in the elongate hemlock scale *Fiorinia externa*; Dybdahl and Kane 2005; Preisser et al. 2008), or from phenotypic plasticity (e.g., in invasive springtail species, in the Mediterranean fruit fly *Ceratitis capitata*; Chown et al. 2007; Nyamukondiwa et al. 2010). Given our care to avoid acclimation as a possible alternative explanation (even if maternal effect has not been ruled out) through our common-garden experiment, it is likely that the specific abiotic tolerance of populations present in human-modified habitats arose as local adaptation within the native range and is not the product of phenotypic plasticity. The relative extent of phenotypic plasticity of native and introduced populations in thermal traits remains to be explored, however, as this may contribute to the successful establishment of populations in dramatically different climatic areas (e.g., the recent invasion of *W. auropunctata* in Israel).

These findings highlight the importance of selective processes due to human activities within the native range. The switch from heat-intolerant sexual populations confined to natural moist and cool habitats to heat-tolerant clonal populations establishing in hot and dry human-modified habitats is probably a first step toward invasive success, as it allows the species to gain access to new resources and to new transport opportunities, specific to human areas. These results are consistent with the ‘two-step’ scenario of invasion process put forward by Foucaud et al. (2010), which is largely based on the potential of native populations to evolve under direct human pressure on landscape properties (see also Hufbauer et al. 2012). It is worth noting, however, that the current study does not strictly demonstrate the ‘two-step’ scenario in the case of the thermotolerance adaptation of *W. auropunctata* populations. To definitively prove this ‘two-step’ scenario, one would additionally require the demonstration of direct genetic relationships between tested native and introduced populations, which was not the case in the present study. Given the strong genetic structure found in native populations at a small geographic scale (Foucaud et al. 2009), a strict demonstration of the two-step scenario for upper thermal limits may prove difficult using *W. auropunctata* as a model species.

The two-step scenario of prior adaptation in the native range before successful long- distance introduction has however been demonstrated in a recent study of the evolution of cold tolerance in native and introduced populations of *W. auropunctata* (Rey et al. 2012). This study showed that the Israeli population, thriving in the colder Mediterranean climate, derived from cold-adapted native populations of Argentina. Most importantly, studies of both upper and lower thermal limits in *W. auropunctata* showed a similar pattern of local adaptation within the native range before dispersal (Rey et al. 2012; this study). These results call for a wider appreciation of both native and introduced population variation when investigating the evolution of phenotypic traits during invasions. An important difference between studies of lower and upper thermotolerance in *W. auropunctata* lies in the nature of the associated selection pressure. Indeed, the study of cold thermotolerance in *W. auropunctata* pointed to a naturally occurring local adaptation at the southern margin of the native range as instrumental in explaining the establishment of the species in a temperate area (Rey et al. 2012). On the contrary, this study of upper thermal abilities pointed to the role of human selective pressures as the main instrumental factor of the evolution of locally adapted thermotolerant native populations. While naturally occurring gradients are likely to produce new adaptations that are predictable in geographic and phenotypic spaces, adaptations to human-modified ecosystems appear much less predictable, because of more abrupt ecological variation and their network-like spatial organization over distribution ranges. To our view, these different types of selective pressure are thus not equally predictable and likely offer different challenges for the management of invasive species.

The hypothesis of human selective pressures modifying species within their native range, driving them toward invasive characteristics, is supported by a growing number of studies on various species. For instance, the ant *Tapinoma sessile*, native to America, is showing a striking transition in social system between populations from natural habitats and populations from highly urbanized habitats within its own native range (Buczkowski 2010). The urban populations also developed invasive characteristics within the native range, such as improved demography and ecological dominance. In this species, the role of human disturbance in the form of urbanization has been clearly demonstrated (Buczkowski 2010; Menke et al. 2010). Unsurprisingly given the dispersal opportunities provided by human- dominated landscapes, it has recently been found to form an invasive population in Hawaii (Buczkowski and Krushelnycky 2012). Agricultural activities also cause major changes in the biology of native species (e.g., distribution, migration, dominance, demography), especially with novel perturbation regimes or the introduction of new hosts (Via 1990). In the butterfly *Euphydryas editha*, a host plant shift occurred within 10 years following the introduction by humans of two plant species, *Plantago lanceolata* and *Collinsia torreyi*, during logging and cattle ranching in previous natural habitats (Singer et al. 1993). The reproductive output of *E. editha* has been shown to be much higher on its new hosts (Singer et al. 1993; Singer and Thomas 1996) and, if its potential for migration were not so low (Brussard and Ehrlich 1970), it might have colonized vast areas in which its new hosts were introduced through agriculture (e.g., in California and Nevada). In some cases, host-switching species evolve invasive features, thrive in their human-modified native range and eventually invade remote areas. This was the case for the Colorado potato beetle, *Leptinotarsa decemlineata*, which switched from its historical *Solanum* hosts to *Solanum tuberosum* following its introduction for farming. It subsequently invaded North America, Europe, and Asia to become the most threatening pest of potato crops (Alyokhin et al. 2008).

In another widely studied invasive ant species, *L. humile*, a recent study has also underlined the significance of both climatic suitability and human disturbance as key determinants of invasion success (Roura-Pascual et al. 2011). It is however important to note that other factors such as the level of biotic interactions can still be relevant to explain some local distribution in *W. auropunctata* (Le Breton et al. 2007), as well as in *L. humile* (Roura-Pascual et al. 2011).

### Effect of body size on thermotolerance

In some ant species, differences in thermotolerance can be accounted for by differences in worker body size (Kaspari 1993; Clemencet et al. 2010). For instance, large *Cataglyphis velox* workers withstand temperatures 6–8°C higher than those tolerated by smaller workers (Cerda and Retana 1997). However, morphological measurements indicated that this was not the case in *W. auropunctata,* as workers of both heat-tolerant populations (i.e., established in human-modified habitats in both the native and the introduced range) and heat-intolerant populations (i.e., established in natural habitats) had bodies of similar size. McGlynn (1999) and Mikheyev and Mueller (2007) found that workers from introduced populations were smaller than those of native populations, based on a wide geographic sampling range. Additional Student's *t-*tests on pairs of population types revealed only a non- significant trend of this type in our own data set (results not shown). Most importantly, the goal of our morphological analysis was to assess the possible relationship between body size and levels of thermotolerance in population samples for which we had thermotolerance data. In that respect, we found no significant association between these two variables. Thermotolerance in *W. auropunctata* is therefore probably achieved through physiological, biochemical, or behavioral processes, or through a combination of such processes, rather than through an increase in body size. For example, in the small workers of thermophilic *C. rosenhaueri*, thermotolerance results partly from an increase in metabolic rate and a decrease in cuticular water loss, together with differences in body posture (Cerda and Retana 2000).

Physiological or other processes explaining the polymorphism in thermotolerance remain to be investigated in *W. auropunctata*. In addition to other experimental investigations, direct approaches using functional loci could be envisaged to study the genetic determinism of thermotolerance in *W. auropunctata*. Our thermotolerance assays could thus be complemented with transcriptomic data, through the use of dedicated microarrays or investigation of the expression patterns of candidate genes, such as genes from the heat-shock protein family (e.g. Dahlgaard et al. 1998; Fangue et al. 2006).

### Alternative adaptive scenarios

Our microsatellite data set showed that the three tested introduced populations were genetically closer to the native populations investigated here for thermotolerance than to any other sampled native populations, but failed to prove close genetic relationships between tested native and introduced populations. Some unsampled native populations are thus probably the source(s) for the three introduced populations studied here. If such unsampled native source populations would not show any increased thermotolerance level, one would then favor a scenario in which adaptation occurred in the introduced range, after long-distance dispersal. However, we advocate that the present knowledge of the biology of *W. auropunctata* renders this alternative scenario far less parsimonious than the scenario of prior adaptation in the native range, before long-distance dispersal.

First, whatever the precise origin of the introduced populations, our results showed that a phenotypic shift in thermotolerance occurred within the native range, most likely through a local adaptation process. The close genetic relationship between the native populations from natural and human-modified habitats tested in this study is undisputed (Foucaud et al. 2007, 2009). As a consequence, we demonstrated here that, at least, prior adaptation can occur in the native range of *W. auropunctata*. Any hypothetical scenario involving adaptation after long-distance dispersal would hence also have to consider the simultaneous occurrence of an adaptive shift within the native range, before long-distance dispersal.

Second, it is important to note that thermotolerance is not the only phenotypic trait for which it has been demonstrated that a shift occurred within the native range of *W. auropunctata* between natural versus human-modified populations, and that native and introduced populations from human-modified habitats were phenotypically closer to each other than to natural populations from the native range. As a matter of fact, ecological dominance (Orivel et al. 2009) and reproductive system polymorphism (Foucaud et al. 2009) follow the same pattern. Therefore, as more traits accumulate to reveal patterns of phenotypic shifts occurring in the native range and later observed overall the introduced range, it becomes more parsimonious to consider that multiple adaptations occurred at most a few times within the native range prior to long-distance dispersal than independently for multiple traits in multiple introduced populations after long-distance dispersal.

## Conclusions

This study highlights the importance of human land use in bringing about major contemporary evolutionary changes within the native range of species. Those adaptations to human habitats in the native range might constitute prior adaptations when introduced in remote human habitats, often showing little variability. This human-induced process may hence ultimately pave the way for the emergence of new bioinvaders. Human modifications of ecosystems are usually substantial, so most species may fail to cross such large valleys in the adaptive landscape (see Lenormand 2002; Ravigné et al. 2009; Ronce and Kirkpatrick 2001 for theoretical studies on this subject). However, *W. auropunctata* provides a striking example of a species that eventually crossed such a valley within its native range, thereby gaining access to new resources and new areas. This may not be an uncommon process, considering that most of the worst cases of biological invasion are committed by human commensals (Ehrlich 1989; Lowe et al. 2000). More empirical studies within the native and introduced ranges of invaders are needed to draw more general and firmer conclusions on the relative contribution of prior adaptations (with a special emphasis on human habitats) and post- introduction evolutionary events (e.g., multiple introductions, purging of deleterious mutations through moderate bottlenecks, ‘bridgehead’ effect, hybridization; Abbott et al. 2003; Bossdorf et al. 2005; Durka et al. 2005; Ellstrand and Schierenbeck 2000; Facon et al. 2011, 2008; Lombaert et al. 2010) to the success of invasive species (Lee and Gelembiuk 2008; van Kleunen et al. 2010; Hufbauer et al. 2012). This latter point is crucial to help building managing policies to prevent invasions worldwide.

## Supporting Information

**Figure S1.** Preliminary experimental results for rates of worker mortality induced by thermal and hygrometric stress in two native populations of *W. auropunctata*.

**Figure S2.** Measures of genetic differentiation between populations from the native and introduced range of *W. auropunctata*.

**Figure S3.** Mortality rates of workers from each of the sampled populations over all tested thermal and hygrometric conditions.

**Figure S4.** Thermotolerance of workers from each sampled populations for each set of thermal and hygrometric conditions tested.

**Figure S5.** Relationship between mean thermotolerance and mean body size of workers from six worldwide *W. auropunctata* populations.

**Data S1.** Data from the main thermotolerance experiment & body size measurements.
