# Supplementary material for: Thermotolerance adaptation to human-modified habitats occurs in the native range of the invasive ant Wasmannia auropunctata before long-distance dispersal
Source: Evol Appl. 2013 Mar 11;6(4):721–34. doi: 10.1111/eva.12058 (PMC3684750; doi:10.1111/eva.12058)

SUPPORTING INFORMATION

SUPPORTING FIGURE 1: Preliminary experimental results for rates of worker mortality induced by thermal and hygrometric stress in two native populations of *W. auropunctata.*

(A) Mortality rates of one native clonal population from a human-modified habitat (Ker) and one native sexual population from a natural habitat (M7) to various abiotic conditions in a preliminary experiment. (B) Sampling size in number of Petri dishes of 10 workers for each temperature and humidity combination. The color scale represents different ranges of worker mortality rate from low (green) to high (red). Untested conditions are indicated by empty, gray cells. Three out of the four temperature-humidity conditions tested in the main experiments (except 39°C-70%) are represented in bold cells.

A

Relative humidity	Temperature													
	25°C		30°C		34°C		36°C		38°C		40°C		42°C	
	Ker	M7	Ker	M7	Ker	M7	Ker	M7	Ker	M7	Ker	M7	Ker	M7
100%	0.1	0	0	0			0	0	0.03	0	0.37	0.43	1	1
80-85%			0	0	0.01	0.02	0.09	0.08	0.07	0.12	0.56	0.73	1	1
70-75%	0	0	0	0	0.01	0.03	0.05	0.08	0.12	0.24	**0.69**	**0.83**	1	1
60-65%	0	0.01	0	0.01	0.03	0.03	0.03	0.1	**0.29**	**0.48**	0.93	0.98	1	1
50-55%	0	0.01	0	0.03	0	0.03	**0.15**	**0.33**	0.52	0.72	0.95	0.99	1	1
35%	0	0	0.03	0.055	0.23	0.39	0.42	0.57	0.98	1				
25%					0.19	0.48								

0 - 0.1
0.1 - 0.4
0.4 - 0.6
0.6 - 0.9
0.9 - 1

B

Relative humidity	Temperature														
	25°C		30°C		34°C		36°C		38°C		40°C		42°C		Total
	Ker	M7	Ker	M7	Ker	M7	Ker	M7	Ker	M7	Ker	M7	Ker	M7	
100%	4	3	3	5			10	10	9	8	32	36	12	12	144
80-85%			6	5	10	10	26	30	35	40	30	30	10	10	242
70-75%	10	10	10	9	10	10	39	39	39	40	49	50	10	10	335
60-65%	10	10	10	8	10	10	29	30	69	70	30	30	10	10	336
50-55%	10	10	9	10	10	10	29	29	26	25	11	9	10	10	208
35%	10	10	10	10	10	10	30	30	10	10					140
25%					10	10									20
Total	44	43	48	47	60	60	163	168	188	193	152	155	52	52	1425

SUPPORTING FIGURE 2: Measures of genetic differentiation between populations from the native and introduced range of *W. auropunctata*.

(A) Matrix of pairwise *F*st values (θ, Weir & Cockerham 1984) between worldwide populations of *W. auropunctata*. Populations used in the present study for the thermotolerance experiments are in bold. Other worldwide populations were taken from Foucaud et al., 2010. Pairwise *F*st estimations between the three introduced clonal populations used in this study and native French Guianese sexual and clonal populations, introduced clonal populations, native Brazilian and Costa Rican populations were highlighted in dark orange, light orange, blue, purple and red, respectively. Populations names are noted as follows: Cub = Cuba, Gua = Guadeloupe, Domr = Dominican Republic, Dom = Dominica, Coc = Cocos Islands, Gal = Galapagos, Sol = Solomons, Aus = Australia, Van = Vanuatu, Haw = Hawaii, Bra = Brazil and Cric = Costa Rica. (B) Mean pairwise *F*st estimations between the three introduced clonal populations used in the present study and five types of native and introduced populations of *W. auropunctata*. The five types of populations include: native sexual populations of French Guiana (FG sexual), native clonal populations of French Guiana (FG clonal), native Brazilian populations (Brazil), native Costa Rican populations (Costa Rica) and worldwide introduced clonal populations (Intro. Range).

A

	M3-F	M7	M11	KER	PI41	P2-1	P2-2	CAY	NCQ0	CAM	FL	Cub 1	Cub 2	Gua 1	Gua 2	Gua 3	Domr	Dom	Coc	Gal	Sol	Aus	Van	Haw	Bra 1	Bra 2	Bra 3	Bra 4	Bra 5	Bra 6	Cric 1	Cric 2
**M7**	0.174																															
**M11**	0.072	0.136																														
**KER**	0.211	0.245	0.198																													
**PI41**	0.253	0.289	0.251	0.138																												
**P2-1**	0.226	0.265	0.226	0.113	0.068																											
**P2-2**	0.287	0.284	0.269	0.271	0.366	0.342																										
**CAY**	0.270	0.279	0.266	0.307	0.342	0.324	0.378																									
**NCQ0**	0.242	0.290	0.234	0.313	0.349	0.326	0.394	0.338																								
**CAM**	0.288	0.358	0.293	0.346	0.397	0.373	0.431	0.383	0.319																							
**FL**	0.238	0.326	0.267	0.312	0.369	0.338	0.395	0.349	0.385	0.407																						
Cub 1	0.299	0.331	0.275	0.314	0.401	0.370	0.438	0.444	0.409	0.500	0.461																					
Cub 2	0.270	0.311	0.247	0.278	0.362	0.320	0.445	0.422	0.422	0.440	0.331	0.504																				
Gua 1	0.239	0.280	0.241	0.291	0.344	0.329	0.400	0.374	0.333	0.391	0.355	0.457	0.341																			
Gua 2	0.304	0.330	0.278	0.324	0.389	0.356	0.460	0.394	0.371	0.390	0.397	0.511	0.338	0.265																		
Gua 3	0.249	0.305	0.239	0.305	0.365	0.340	0.473	0.447	0.391	0.454	0.447	0.487	0.423	0.338	0.433																	
Domr	0.273	0.314	0.250	0.291	0.357	0.318	0.414	0.392	0.362	0.435	0.343	0.466	0.314	0.245	0.190	0.384																
Dom	0.186	0.231	0.165	0.262	0.331	0.304	0.392	0.361	0.345	0.415	0.355	0.368	0.352	0.299	0.375	0.298	0.357															
Coc	0.282	0.325	0.264	0.372	0.439	0.409	0.487	0.464	0.411	0.498	0.493	0.494	0.498	0.457	0.521	0.488	0.469	0.386														
Gal	0.250	0.249	0.234	0.301	0.372	0.349	0.422	0.369	0.361	0.425	0.375	0.480	0.424	0.324	0.402	0.421	0.312	0.331	0.444													
Sol	0.264	0.269	0.239	0.333	0.389	0.373	0.418	0.381	0.417	0.446	0.415	0.484	0.467	0.391	0.468	0.481	0.446	0.318	0.482	0.393												
Aus	0.220	0.225	0.191	0.296	0.354	0.332	0.393	0.349	0.378	0.412	0.384	0.444	0.408	0.369	0.429	0.422	0.419	0.282	0.453	0.309	0.107											
Van	0.234	0.267	0.219	0.316	0.380	0.354	0.416	0.382	0.356	0.445	0.358	0.486	0.368	0.288	0.394	0.368	0.360	0.320	0.455	0.351	0.354	0.280										
Haw	0.283	0.334	0.264	0.344	0.425	0.391	0.451	0.422	0.450	0.478	0.311	0.532	0.228	0.432	0.463	0.514	0.428	0.387	0.542	0.431	0.474	0.412	0.382									
Bra 1	0.285	0.302	0.261	0.332	0.419	0.390	0.435	0.435	0.426	0.488	0.446	0.462	0.455	0.410	0.483	0.466	0.444	0.327	0.486	0.412	0.439	0.405	0.426	0.481								
Bra 2	0.274	0.290	0.255	0.334	0.419	0.389	0.440	0.408	0.402	0.487	0.440	0.464	0.443	0.402	0.473	0.464	0.444	0.335	0.471	0.413	0.435	0.400	0.417	0.468	0.020							
Bra 3	0.295	0.311	0.278	0.344	0.429	0.403	0.422	0.439	0.425	0.505	0.455	0.492	0.468	0.422	0.494	0.496	0.441	0.343	0.474	0.431	0.444	0.418	0.434	0.496	0.000	0.018						
Bra 4	0.254	0.279	0.237	0.332	0.408	0.376	0.411	0.439	0.410	0.477	0.472	0.490	0.457	0.443	0.483	0.484	0.442	0.353	0.449	0.429	0.449	0.408	0.445	0.489	0.456	0.446	0.462					
Bra 5	0.285	0.338	0.255	0.379	0.452	0.416	0.497	0.486	0.474	0.541	0.479	0.534	0.542	0.518	0.576	0.559	0.541	0.381	0.543	0.514	0.472	0.430	0.474	0.568	0.485	0.481	0.504	0.477				
Bra 6	0.308	0.347	0.281	0.392	0.455	0.426	0.501	0.479	0.440	0.516	0.513	0.547	0.548	0.498	0.551	0.548	0.495	0.389	0.492	0.463	0.527	0.474	0.510	0.586	0.487	0.470	0.513	0.431	0.523			
Cric 1	0.274	0.326	0.261	0.382	0.450	0.420	0.471	0.467	0.462	0.517	0.488	0.544	0.527	0.497	0.551	0.511	0.503	0.379	0.497	0.476	0.503	0.452	0.486	0.530	0.479	0.473	0.499	0.421	0.536	0.533		
Cric 2	0.243	0.287	0.230	0.322	0.381	0.351	0.442	0.408	0.380	0.435	0.451	0.455	0.442	0.392	0.436	0.403	0.424	0.317	0.349	0.369	0.442	0.370	0.387	0.485	0.419	0.408	0.431	0.369	0.466	0.432	0.426	
Cric 3	0.232	0.254	0.211	0.330	0.387	0.361	0.436	0.416	0.388	0.404	0.443	0.468	0.426	0.375	0.440	0.441	0.413	0.308	0.372	0.390	0.389	0.341	0.382	0.452	0.401	0.391	0.404	0.377	0.460	0.462	0.341	0.311

B

	FG sexual	FG clonal	Brazil	Costa Rica	Intro. Range
NCQ0	0.255	0.344	0.430	0.410	0.385
CAM	0.313	0.386	0.502	0.452	0.441
FI	0.277	0.353	0.467	0.461	0.386

SUPPORTING FIGURE 3: Mortality rates of workers from each of the sampled populations over all tested thermal and hygrometric conditions.

Diamonds indicate means, blocks and horizontal bars indicate 50% and 95% percentiles, respectively.

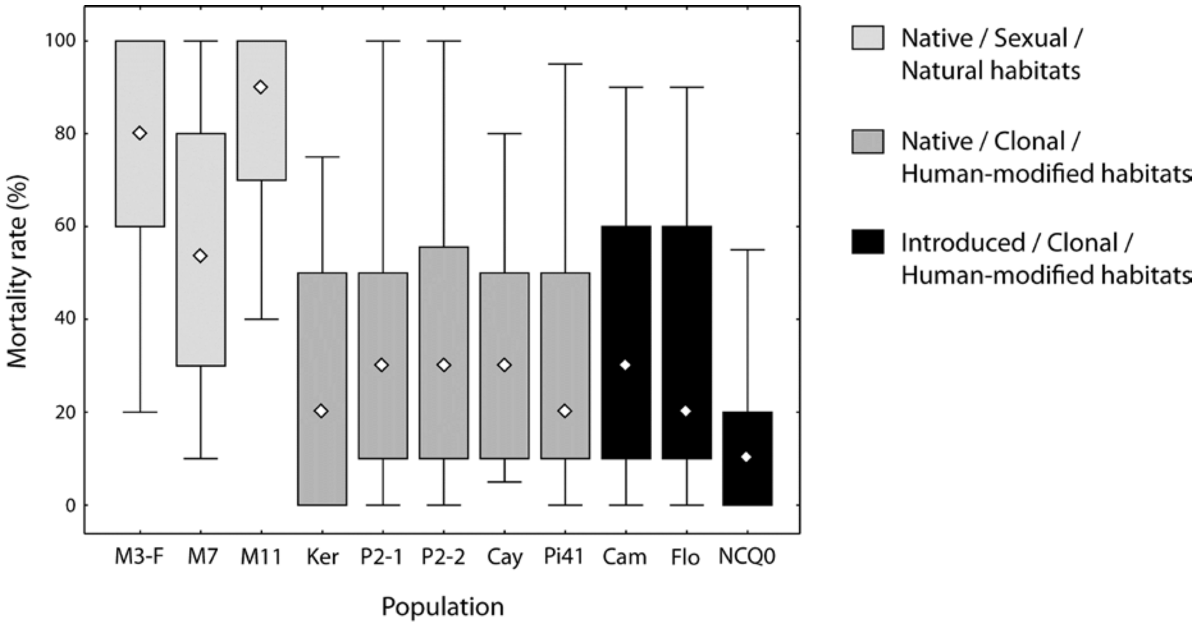

SUPPORTING FIGURE 4: Thermotolerance of workers from each sampled populations for each set of thermal and hygrometric conditions tested.

Diamonds indicate means, blocks and horizontal bars indicate 50% and 95% percentiles, respectively.

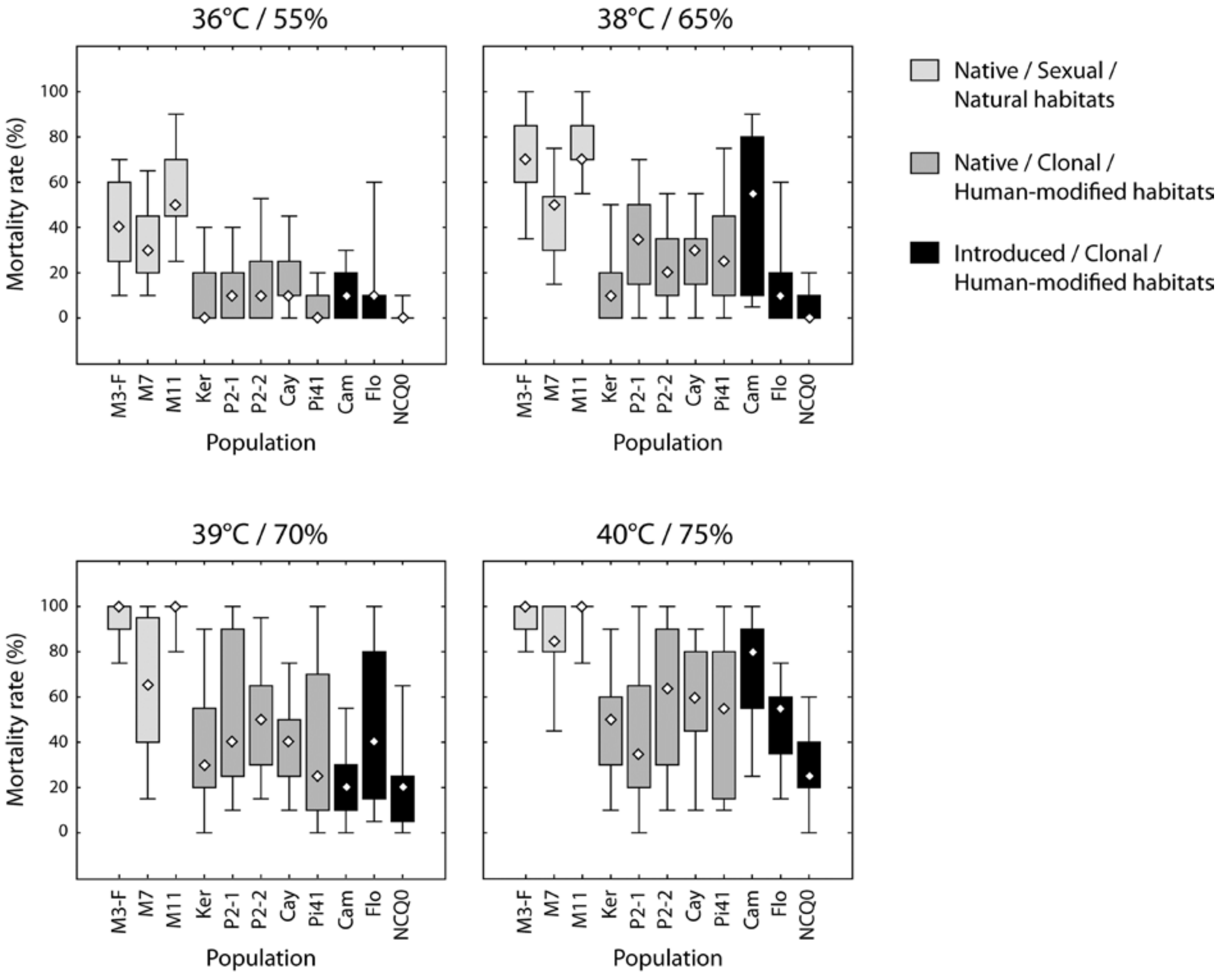

SUPPORTING FIGURE 5: Relationship between mean thermotolerance and mean body size of workers from 6 worldwide *W. auropunctata* populations.

Errors bars indicate standard deviation.

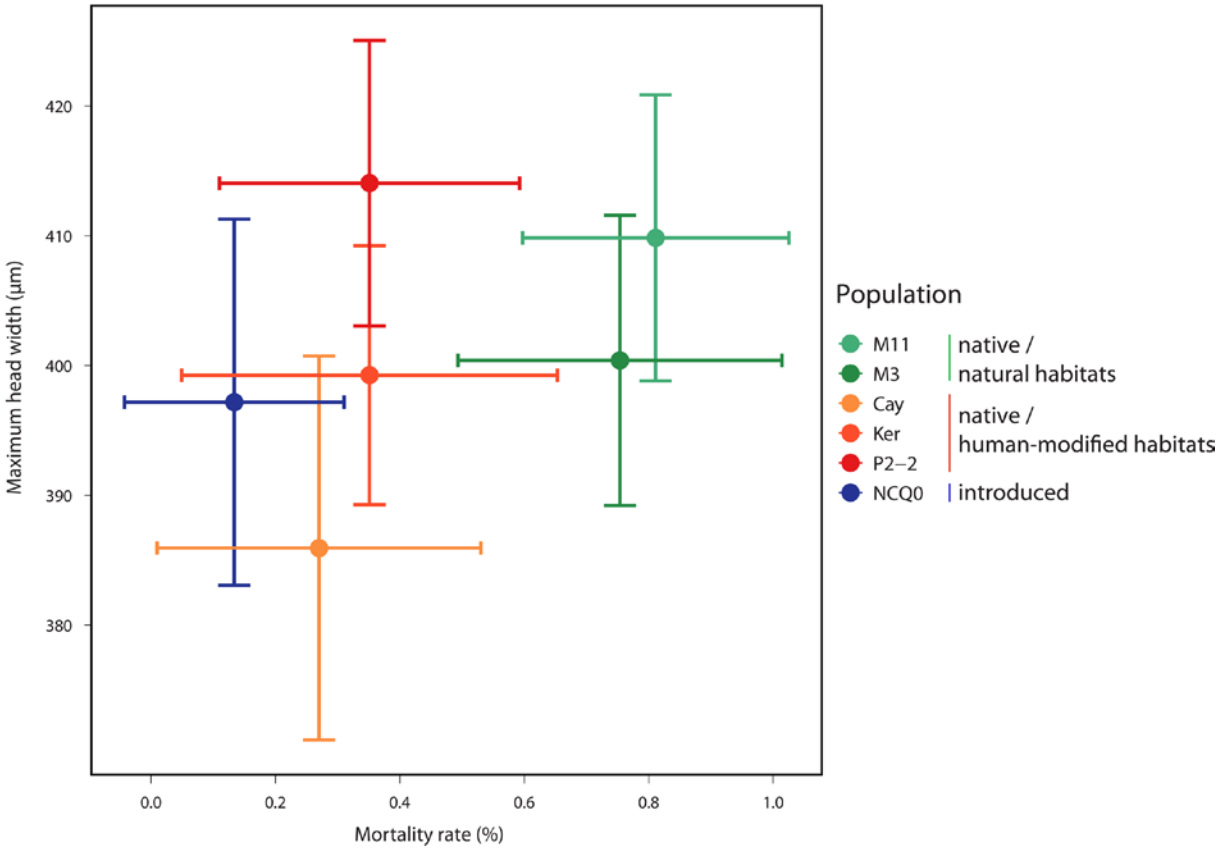

Supplement: Supplementary file 1 [file eva0006-0721-SD1.pdf]
